# Patterns and Influencing Factors of eHealth Tools Adoption Among Medicaid and Non-Medicaid Populations From the Health Information National Trends Survey (HINTS) 2017-2019: Questionnaire Study

**DOI:** 10.2196/25809

**Published:** 2021-02-18

**Authors:** Xin Yang, Ning Yang, Dwight Lewis, Jason Parton, Matthew Hudnall

**Affiliations:** 1 Institute of Data and Analytics The University of Alabama Tuscaloosa, AL United States; 2 Department of Information Systems, Statistics, and Management Science Culverhouse College of Business The University of Alabama Tuscaloosa, AL United States; 3 Department of Management Culverhouse College of Business The University of Alabama Tuscaloosa, AL United States

**Keywords:** Medicaid program, eHealth, internet access, digital divide, health information technology

## Abstract

**Background:**

Evidence suggests that eHealth tools adoption is associated with better health outcomes among various populations. The patterns and factors influencing eHealth adoption among the US Medicaid population remain obscure.

**Objective:**

The objective of this study is to explore patterns of eHealth tools adoption among the Medicaid population and examine factors associated with eHealth adoption.

**Methods:**

Data from the Health Information National Trends Survey from 2017 to 2019 were used to estimate the patterns of eHealth tools adoption among Medicaid and non-Medicaid populations. The effects of Medicaid insurance status and other influencing factors were assessed with logistic regression models.

**Results:**

Compared with the non-Medicaid population, the Medicaid beneficiaries had significantly lower eHealth tools adoption rates for health information management (11.2% to 17.5% less) and mobile health for self-regulation (0.8% to 9.7% less). Conversely, the Medicaid population had significantly higher adoption rates for using social media for health information than their counterpart (8% higher in 2018, *P*=.01; 10.1% higher in 2019, *P*=.01). Internet access diversity, education, and cardiovascular diseases were positively associated with health information management and mobile health for self-regulation among the Medicaid population. Internet access diversity is the only factor significantly associated with social media adoption for acquisition of health information (OR 1.98, 95% CI 1.26-3.11).

**Conclusions:**

Our results suggest digital disparities in eHealth tools adoption between the Medicaid and non-Medicaid populations. Future research should investigate behavioral correlates and develop interventions to improve eHealth adoption and use among underserved communities.

## Introduction

Though progress has been made with improving the “digital divide” in the United States, disparities in internet access remain among underserved populations such as the elderly and individuals in lower socioeconomic classifications [1]. This digital divide can have profound implications on public health because internet access is hypothesized as a “super-determinant” of health [2]. One primary way by which the internet may improve health outcomes is through the adoption of electronic health (eHealth). eHealth is the application and integration of information and communication technologies (ICT) to improve patients' health care, enable more reliable connections between patients and health providers, and reduce health disparities [3,4].

Standard eHealth tools used by consumers include the utilization of electronic health records (EHRs), health-related social media channels to seek or share health information, and online patient-provider communication (OPPC) channels such as online portals, email, and social media [5]. Recent advancements in information technology (IT) expanded the adoption of eHealth tools to smart devices such as mobile health tools (eg, smartphone apps, wearable devices such as Fitbit, and smartwatches) [6] and virtual assistants (eg, Amazon Alexa) for health information [7].

Findings from the literature suggest that eHealth adoption is associated with health-protective outcomes among individuals. Previous studies purport that health IT adoption among individuals promotes healthy behaviors such as fruit and vegetable intake [8,9]. In addition, patients' use of online eHealth tools to communicate with providers is associated with increased access to health-related information, reduction of health care expenditures, improved emotional support, and enhanced clinical management and self-care [10-12]. Available findings also suggest that social media use among patients is associated with a positive impact on facilitating self-care [13-16]. With respect to prescription medication adherence, the use of eHealth tools to refill prescribed drugs is positively correlated with statin adherence among patients experiencing cardiovascular disease [17,18]. The US Department of Health and Human Services (HHS) recognizes the value that internet access and eHealth tools have on health outcomes, so they developed national goals in Healthy People 2020 to bolster access [19]. The HHS also created the Office of the National Coordinator, which has been advancing consumer eHealth via incentive programs, supporting developers to build eHealth tools, and strengthening trust and protecting privacy in health information technology (HIT) tools [20].

There is an emerging body of literature reporting the factors that influence eHealth tools adoption by consumers. Attributes related to geography, socioeconomic status, and gender are suggested to be associated with the utilization of eHealth tools. Geographic disparities in health IT uses were found between rural and urban residents. Specifically, rural residents were less likely to access online medical records [21]. Higher levels of education contribute to managing electronic personal health information (ePHI) online [22], OPPC [5], and use of health IT for self-regulation [8], which indicates that having a higher education is associated with better eHealth adoption and utilization. Gender has a mixed effect on eHealth tools adoption, depending on the scenario. Men with a risk of cardiovascular disease (CVD) have a higher likelihood of using wearable devices than people without CVD risk. However, there does not appear to be a difference among women [6]. Females had a significantly higher probability of using ePHI online management compared to males [22,23], but there is no significant difference in online patient-provider communication [5,23]. Income level was reported to be positively associated with eHealth sum score, a composite score representative of using technology to access health care [22,24].

Given that this study's focus population are Medicaid enrollees, a primary area of interest is the impact education and income have on individuals qualifying for this service. The inverse relationship between socioeconomic status and adverse health outcomes is well documented in public health literature. In the United States, increased household income is associated with longer life expectancy [25]. Though the causative nature of the relationship between cardiovascular disease and socioeconomic factors is yet to be fully understood, being classified in a lower socioeconomic stratum is linked to poor health outcomes such as hypertension, diabetes mellitus, and coronary heart disease [26]. Due to factors such as these and the unique challenges that financial hardship has on access to medical care [27], the United States government amended provisions in the Social Security Act, which established a health insurance program for impoverished citizens (ie, Medicaid). Findings with respect to internet access [28] parallel those of the underserved in the United States. Moreover, not to imply that being on Medicaid causes poor health outcomes, there is literature suggesting that Medicaid beneficiaries have poorer health outcomes that can lead to long-term cardiovascular complications compared to privately insured individuals [29].

Acknowledging the fact that one must have internet access to utilize eHealth tools and that national efforts should be made in the US to increase accessibility, there remain certain sectors of the population that are limited in this capacity. Nevertheless, given considerations of the potential health benefits that eHealth adoption may confer and current health disparities among Medicaid beneficiaries, a question we propose is “Are eHealth adoption rates among Medicaid beneficiaries comparable to those of people who are not on Medicaid?” To our best knowledge, no study has portrayed a comprehensive picture of eHealth tools adoption among adult Medicaid beneficiaries, an underserved population. Medicare and Medicaid EHR Incentive Programs have been promoting HIT adoption among providers and achieved a significant impact [20]. However, how Medicaid beneficiaries adopt eHealth tools was mostly unknown nationally. Due to the unique characteristics of Medicaid beneficiaries, the factors of eHealth adoption among the adult Medicaid population might also have different effects compared to those for the non-Medicaid community. Analyses from this study are focused on contrasting these characteristics between the two groups to provide research that may lead to health policy implications that positively impact America's underserved.

In this study, we use 3 years of the US Health Information National Trends Survey (HINTS) data to investigate the following research objectives (RO): RO1, comparison of eHealth tools adoption among Medicaid beneficiaries versus non-Medicaid insured individuals from 2017 to 2019; RO2, the association of Medicaid status with eHealth adoption; and RO3, compared to the non-Medicaid population, what factors are associated with increased eHealth adoption among the Medicaid population. To examine these questions, we assessed seven domains of eHealth tools utilization outcomes that covered a variety of HIT tools such as secure internet portals, EHRs, patient-provider email messaging, mobile health apps, and wearable devices as described in the report [20].

## Methods

### Data and Sample

We extracted 3 waves of HINTS data that include the calendar years of 2017, 2018, and 2019. HINTS is a two-stage, cross-sectional, and nationally representative survey sampled from noninstitutionalized adults in the United States [30]. Since eHealth tools adoption is mainly dependent on internet access, HINTS 2017-2019 data operationalize internet access by participants who self-reported access to the internet in the following ways: regular dial-up telephone line, broadband, WiFi, and 3G or 4G cellular network. With this criterion, we excluded respondents who reported no internet access and obtained a sample size of 2513 respondents in 2017, 2704 respondents in 2018, and 4264 respondents in 2019.

### Dependent Variables

We constructed seven primary outcomes that represented a variety of eHealth tools adoption and utilization behaviors. OPPC [5] is composed of three items: (1) used electronic means to communicate with a doctor, (2) whether tablet or smartphone helped discuss with health care providers, and (3) used online medical record to securely message health care providers. Health information management (HIM) [23] is composed of two items: (1) used electronic means to track health care charges and (2) used electronic means to look up medical test results. Mobile health for self-regulation (MHSR) [8] is composed of three items: (1) have apps related to health and wellness, (2) used electronic medical device to track health, and (3) used tablet or smartphone to help track progress on a health-related goal. Social media for health information (SMHI) [23] is composed of three items: (1) used internet to participate in an online forum or support group, (2) used internet to watch a health-related video on YouTube, and (3) used internet to share health information on social networking sites.

Sharing information [31] is composed of four items: (1) shared health information with a health professional via electronic device or smartphone, (2) electronically sent medical information to another health care provider, (3) electronically sent medical information to a family member or another person, and (4) electronically sent medical information to a service or app that helps manage and store health information. Buy or refill medicine (BRM) [32] is composed of two items: (1) used online medical record to request a refill of medications and (2) used electronic means to buy medicine or vitamins online. Decision making [31] is composed of two items: (1) used online medical record to help you make a decision about how to treat an illness or condition and (2) tablet or smartphone helped make a decision about how to treat an illness or condition. The definitions of each outcome are shown in Multimedia Appendix 1. The composite scores were constructed by summing their dichotomous items with responses “No” and “Yes” converted to 0 and 1, respectively, except that scores higher than 1 in SMHI were recoded to 2 due to sparsity of participants who share information via multiple channels.

### Key Independent Variables and Covariates

Medicaid status is determined by participants' self-reported Medicaid insurance plan. Internet access diversity score was measured by whether participants had only one channel or multiple channels to access the internet. CVD risk was defined by having at least one of three conditions: hypertension, a heart condition, or diabetes. Depression was defined by whether respondents had been told they have depression or an anxiety disorder by a doctor or health professional. Demographic and socioeconomic variables included gender, age, race or ethnicity, education, residency, US Census region, and annual household income (Multimedia Appendix 2).

### Statistical Analysis

We used descriptive statistics (frequencies and proportions) to demonstrate sample characteristics and eHealth tools adoption distribution patterns of HINTS data from 2017 to 2019. Pearson chi-square tests were performed to examine the association between Medicaid insurance status and other variables, including demographic, socioeconomic status, and eHealth outcomes. We considered a *P* value of less than or equal to .05 to be statistically significant. To answer RO2, we conducted ordinal logistic regressions for each of the seven eHealth outcomes using Medicaid status as the factor of primary interest. Base models included survey year as the only covariate, while adjusted models included the variables of age group, gender, education, Census region, residency, income, and internet access diversity. To answer RO3 and provide a comparison of detailed factor profiles in each population, we conducted ordinal logistic regression for each outcome within the Medicaid sample and non-Medicaid sample independently. In addition to demographic and socioeconomic factors, we included cardiovascular disease, depression, and internet access diversity as independent variables in the models. All statistical analyses except frequencies were conducted through jackknife weight procedures to represent national population-level estimates and obtain more precise confidence intervals. All analyses were performed in SAS 9.4 (SAS Institute).

## Results

Table 1 presents the sample characteristics of both Medicaid and non-Medicaid population from 2017 to 2019. Compared to the people who were not enrolled in the Medicaid program, the Medicaid population is more likely to be young, belong to an underrepresented minority (Hispanic and non-Hispanic Black), and have low education and low family income. Generally, there is no significant difference in the distributions of residency between Medicaid and non-Medicaid populations. Interestingly, the proportions of people who have multiple ways to access the internet were not significantly different between Medicaid (68.6%) and non-Medicaid participants (67.6%), though the proportion of Medicaid beneficiaries who have no internet access at all was significantly higher than that of the non-Medicaid population (Multimedia Appendix 3).

**Table 1:** Demographic characteristics of sample from 3 iterations, 2017-2019, by Medicaid insurance status.

Variable		Medicaid (N=1087)	Non-Medicaid (N=8394)		*P* value^a^
**Gender, n (%)**				.01	
	Male	349 (42.1^b^)	3545 (50.0)		—^c^
	Female	716 (57.9)	4738 (50.0)		—
**Education, n (%)**				<.001	
	Less than high school	114 (11.3)	193 (4.0)		—
	High school graduate	243 (29.7)	1039 (17.6)		—
	Some college	445 (44.6)	2413 (39.2)		—
	College graduate or higher	262 (14.4)	4621 (39.2)		—
**Age group (years), n (%)**				<.001	
	18-24	76 (20.3)	225 (9.4)		—
	25-44	328 (36.1)	2108 (32.9)		—
	45-64	475 (36.4)	3384 (41.9)		—
	65+	181 (7.2)	2514 (15.8)		—
**Race, n (%)**				<.001	
	Hispanic	223 (22.7)	929 (13.5)		—
	Non-Hispanic White	450 (51.3)	5439 (69.1)		—
	Non-Hispanic Black	214 (15.8)	881 (8.9)		—
	Non-Hispanic Other	66 (5.1)	274 (2.9)		—
	Non-Hispanic Asian	48 (5.1)	350 (5.5)		—
**Household income (US $), n (%)**				<.001	
	Less than $20,000	528 (46.8)	564 (8.0)		—
	$20,000 to <$35,000	200 (21.7)	801 (8.6)		—
	$35,000 to <$50,000	129 (13.8)	991 (13.1)		—
	$50,000 to <$75,000	80 (10.4)	1615 (20.9)		—
	$75,000 or more	70 (7.2)	3666 (49.3)		—
**Residency, n (%)**				.82	
	Urban	970 (86.8)	7435 (87.2)		—
	Rural	117 (13.2)	959 (12.8)		—
**Census region, n (%)**				<.001	
	Northeast	194 (17.8)	1252 (17.5)		—
	Midwest	193 (21.2)	1503 (20.9)		—
	South	365 (29.5)	3657 (38.6)		—
	West	335 (31.5)	1982 (23.0)		—
**Internet access diversity, n (%)**				.67	
	One way to access internet	444 (31.2)	3289 (32.4)		—
	More than one way to access	643 (68.8)	5105 (67.6)		—

^a^Chi-square tests were conducted to obtain *P* values adjusted for sampling weights.

^b^Percentages were weighted by jackknife weighting methods to represent US population-level estimates.

^c^Not available.

We investigated eHealth tools adoption rates among Medicaid and non-Medicaid populations. Table 2 shows the frequencies and weighted proportions of respondents by eHealth tools adoption scores. The higher scores suggest a better adoption and utilization of eHealth tools. Chi-square test results suggested no statistically significant disparity between Medicaid and non-Medicaid respondents for sharing information, decision making, OPPC, or BRM, with exceptions for BRM in 2017 and OPPC in 2018. In the Medicaid population, the respondents were densely distributed in the score “0” of HIM with a range of 51.1% to 64.9%, which was 11.2% to 17.5% higher than that of the non-Medicaid population (*P*<.001). In 2018, 42.1% of Medicaid respondents had a 0 score for MHSR, which is 7.7% higher than non-Medicaid (*P*=.02). However, in 2019, the proportion of Medicaid respondents who reported a MHSR score of 0 dropped to 27.9%, only 0.8% higher than that of non-Medicaid. The proportions of Medicaid respondents were 7.8% and 9.4% less in scores 2 and 3 for MHSR compared to those of the non-Medicaid population (*P*<.001). Contrary to HIM and MHSR, Medicaid respondents had significantly higher SMHI adoption rates than non-Medicaid respondents in 2018 (8% higher, *P*=.01) and 2019 (10.1% higher, *P*=.01).

**Table 2:** The frequency and weighted prevalence of eHealth tools adoption among Medicaid and non-Medicaid populations from 2017 to 2019.

Variable			2017						2018						2019				
			M^a^ (N=285)		NM^b^ (N=2228)		*P* value^c^		M (N=299)		NM (N=2405)		*P* value		M (N=503)		NM (N=3761)		*P* value
**SI^d^ score, n (%)**							.94						.81						.98
	0	221 (81.2)		1738 (80.3)		—^e^		221 (80.5)		1816 (78.5)		—		363 (75.3)		2804 (75.9)		—	
	1	55 (16.2)		418 (17.3)		—		60 (15.7)		493 (18.0)		—		114 (20.5)		799 (19.9)		—	
	2	7 (2.6)		70 (2.3)		—		18 (3.8)		88 (3.5)		—		24 (4.3)		155 (4.3)		—	
**DM^f^ score, n (%)**							.69						.54						.32
	0	162 (60.4)		1429 (62.6)		—		168 (60.9)		1400 (58.0)		—		250 (49.2)		2089 (56.0)		—	
	1	110 (37.4)		696 (33.9)		—		105 (32.4)		851 (36.9)		—		204 (43.1)		1328 (37.2)		—	
	2	10 (2.2)		85 (3.5)		—		22 (6.7)		137 (5.1)		—		38 (7.7)		303 (6.8)		—	
**HIM^g^ score, n (%)**							.01						<.001						.01
	0	172 (61.2)		986 (44.3)		—		176 (64.9)		1029 (47.4)		—		261 (51.1)		1435 (39.9)		—	
	1	78 (30.8)		688 (31.0)		—		78 (25.9)		690 (26.6)		—		142 (29.4)		1182 (31.3)		—	
	2	33 (8.0)		549 (24.7)		—		41 (9.2)		663 (26.0)		—		100 (19.5)		1136 (28.8)		—	
**BRM^h^ score, n (%)**							<.001						.08						.46
	0	218 (83.0)		1414 (67.0)		—		216 (74.7)		1492 (66.0)		—		307 (58.8)		2082 (56.6)		—	
	1	54 (14.0)		654 (27.0)		—		66 (20.9)		720 (27.6)		—		153 (33.4)		1235 (32.5)		—	
	2	10 (2.9)		156 (5.9)		—		17 (4.4)		189 (6.4)		—		41 (7.8)		438 (10.9)		—	
**MHSR^i^ score, n (%)**							.07						.02						<.001
	0	117 (44.1)		789 (34.4)		—		121 (42.1)		802 (34.4)		—		142 (27.9)		1039 (27.1)		—	
	1	80 (26.3)		565 (24.4)		—		73 (23.6)		560 (20.1)		—		171 (37.4)		948 (21.0)		—	
	2	54 (18.7)		451 (22.1)		—		67 (22.7)		490 (22.6)		—		99 (16.6)		813 (24.4)		—	
	3	33 (10.9)		421 (19.1)		—		35 (11.6)		529 (22.9)		—		90 (18.1)		960 (27.5)		—	
**SMHI^j^ score, n (%)**							.14						.01						.01
	0	137 (48.0)		1369 (57.7)		—		146 (48.0)		1385 (56.0)		—		224 (43.8)		2163 (53.9)		—	
	1	98 (40.3)		590 (29.2)		—		86 (29.4)		711 (32.2)		—		166 (33.0)		1150 (31.8)		—	
	2	28 (7.2)		201 (9.6)		—		45 (17.2)		214 (9.3)		—		79 (16.5)		320 (10.4)		—	
	3	20 (4.4)		65 (3.5)		—		18 (5.4)		73 (2.5)		—		34 (6.7)		118 (3.9)		—	
**OPPC^k^ score, n (%)**							.21						<.001						.92
	0	155 (53.8)		966 (44.9)		—		159 (62.8)		995 (44.9)		—		217 (38.0)		1349 (39.1)		—	
	1	71 (27.6)		634 (28.7)		—		76 (19.1)		669 (25.7)		—		139 (28.7)		993 (26.0)		—	
	2	37 (10.8)		410 (18.2)		—		33 (9.6)		453 (18.4)		—		89 (20.3)		854 (21.0)		—	
	3	21 (7.8)		215 (8.2)		—		27 (8.6)		276 (11.0)		—		57 (12.9)		562 (13.9)		—	

^a^M: Medicaid population.

^b^NM: non-Medicaid population.

^c^Chi-square tests were conducted to obtain *P* values adjusted for sampling weights.

^d^SI: sharing information.

^e^Not available.

^f^DM: decision making.

^g^HIM: health information management.

^h^BRM: buy or refill medicine.

^i^MHSR: mobile health for self-regulation.

^j^SMHI: social media for health information.

^k^OPPC: online patient-provider communication.

Multimedia Appendix 4 shows that the effects of Medicaid status on BRM and OPPC were attenuated after adjusting multiple covariates. Consistent with Table 2, Medicaid beneficiaries were significantly less likely to use eHealth tools for HIM (OR 0.64, 95% CI 0.50-0.82) and MHSR (OR 0.63, 95% CI 0.53-0.76), but more likely for SMHI (OR 1.35, 95% CI 1.12-1.63). To address RO3, we further assessed the factor effects such as demographic, socioeconomic, health, and internet access on these three eHealth categories in the Medicaid and non-Medicaid populations independently. Table 3 and 4 shows that in the non-Medicaid population, HIM and MHSR were more likely to be adopted by individuals who were female, were well educated, had high-income households, and had more than one source of internet access. Having cardiovascular disease or depression was also significantly associated with HIM and MHSR. Among the non-Medicaid respondents, the SMHI was significantly associated with factors such as gender, race, age, depression, and internet access diversity (Table 5). However, there were fewer factors significantly associated with eHealth tools adoption among the Medicaid respondents. Education (*P*=.03), CVD risk (OR 2.16, 95% CI 1.18-3.93; *P*=.01), depression (OR 1.67, 95% CI 1.07-2.6; *P*=.02), and internet access diversity (OR 2.87, 95% CI 1.55-5.30; *P*=.001) were factors significantly associated with HIM (Table 3). Education (*P*=.02), CVD risk (OR 2.55, 95% CI 1.52-4.3; *P*<.001) and internet access diversity (OR 2.72, 95% CI 1.69-4.38; *P*<.001) were factors significantly associated with MHSR (Table 4). Internet access (OR 1.98, 95% CI 1.26-3.11; *P*=.004) was the only significant factor associated with SMHI (Table 5).

**Table 3:** The odds ratio of predictors for health information management via eHealth tools among Medicaid and non-Medicaid participants.

Variable		Medicaid		Non-Medicaid	
		OR^a^ (95% CI)	*P* value	OR (95% CI)	*P* value
**Year**					
	2017	Ref	.01	Ref	.01
	2018	1.23 (0.68-2.22)	—^b^	0.95 (0.79-1.15)	—
	2019	2.22 (1.29-3.80)	—	1.24 (1.03-1.49)	—
**Gender**					
	Male	Ref	.83	Ref	<.001
	Female	0.95 (0.57-1.58)	—	1.36 (1.15-1.60)	—
**Race**					
	Non-Hispanic White	Ref	.80	Ref	.02
	Hispanic	1.43 (0.72-2.84)	—	0.92 (0.69-1.22)	—
	Non-Hispanic Black	1.31 (0.72-2.37)	—	0.69 (0.51-0.95)	—
	Non-Hispanic Other	0.83 (0.32-2.16)	—	1.10 (0.65-1.86)	—
	Non-Hispanic Asian	1.31 (0.37-4.65)	—	1.59 (1.04-2.44)	—
**Education**					
	Less than high school	Ref	.03	Ref	<.001
	High school graduate	0.84 (0.34-2.09)	—	1.71 (0.95-3.07)	—
	Some college	1.68 (0.68-4.14)	—	2.85 (1.56-5.23)	—
	College graduate or higher	2.33 (0.91-5.92)	—	4.37 (2.48-7.70)	—
**Age group (years)**					
	18-24	Ref	.72	Ref	.06
	25-44	1.58 (0.66-3.80)	—	1.54 (1.01-2.35)	—
	45-64	1.46 (0.55-3.85)	—	1.37 (0.91-2.06)	—
	65+	1.63 (0.55-4.86)	—	1.18 (0.76-1.82)	—
**Census region**					
	Northeast	Ref	.55	Ref	.03
	Midwest	1.36 (0.70-2.66)	—	1.22 (0.95-1.56)	—
	South	1.50 (0.69-3.27)	—	1.21 (0.93-1.58)	—
	West	0.95 (0.46-1.99)	—	1.46 (1.14-1.87)	—
**Residency**					
	Urban	Ref	.90	Ref	.01
	Rural	0.96 (0.49-1.87)	—	0.71 (0.55-0.90)	—
**Household income (US $)**					
	Less than $20,000	Ref	.23	Ref	<.001
	$20,000 to <$35,000	0.85 (0.47-1.55)	—	1.09 (0.69-1.73)	—
	$35,000 to <$50,000	1.76 (0.95-3.25)	—	1.48 (0.89-2.44)	—
	$50,000 to <$75,000	1.40 (0.68-2.91)	—	1.56 (0.96-2.54)	—
	$75,000 or more	1.79 (0.70-4.56)	—	1.94 (1.20-3.13)	—
**Cardiovascular disease**					
	No	Ref	.01	Ref	.01
	Yes	2.16 (1.18-3.93)	—	1.29 (1.07-1.56)	—
**Depression**					
	No	Ref	.02	Ref	<.001
	Yes	1.67 (1.07-2.60)	—	1.41 (1.18-1.68)	—
**Internet access diversity**					
	One way to access internet	Ref	.001	Ref	<.001
	More than one way to access	2.87 (1.55-5.30)	—	1.45 (1.18-1.78)	—

^a^OR: odds ratio.

^b^Not available.

**Table 4:** The odds ratio of predictors for mobile health for self-regulation among Medicaid and non-Medicaid participants.

Variable		Medicaid				Non-Medicaid		
		OR^a^ (95% CI)		*P* value		OR (95% CI)		*P* value
**Year**								
	2017		Ref		.10		Ref	<.001
	2018		1.28 (0.73-2.24)		—^b^		1.18 (0.96-1.44)	—
	2019		1.73 (1.04-2.88)		—		1.49 (1.24-1.80)	—
**Gender**								
	Male		Ref		.21		Ref	<.001
	Female		1.37 (0.83-2.26)		—		1.56 (1.35-1.80)	—
**Race**								
	Non-Hispanic White		Ref		.57		Ref	.07
	Hispanic		1.47 (0.85-2.54)		—		1.14 (0.92-1.42)	—
	Non-Hispanic Black		1.35 (0.57-3.19)		—		1.16 (0.88-1.55)	—
	Non-Hispanic Other		1.55 (0.45-5.27)		—		1.37 (0.97-1.92)	—
	Non-Hispanic Asian		0.78 (0.33-1.81)		—		1.32 (0.86-2.01)	—
**Education**								
	Less than high school		Ref		.02		Ref	<.001
	High school graduate		1.14 (0.44-2.99)		—		0.97 (0.61-1.54)	—
	Some college		0.78 (0.31-1.97)		—		1.74 (1.18-2.57)	—
	College graduate or higher		1.82 (0.65-5.11)		—		2.04 (1.38-3.02)	—
**Age group (years)**								
	18-24		Ref		.37		Ref	<.001
	25-44		0.59 (0.23-1.50)		—		0.94 (0.65-1.36)	—
	45-64		0.47 (0.18-1.26)		—		0.63 (0.43-0.93)	—
	65+		0.45 (0.18-1.15)		—		0.41 (0.28-0.60)	—
**Census region**								
	Northeast		Ref		.99		Ref	.03
	Midwest		1.02 (0.50-2.09)		—		1.23 (0.97-1.55)	—
	South		0.94 (0.46-1.89)		—		1.36 (1.11-1.67)	—
	West		1.03 (0.47-2.30)		—		1.22 (1.01-1.47)	—
**Residency**								
	Urban		Ref		.97		Ref	.51
	Rural		1.01 (0.51-2.03)		—		0.93 (0.75-1.16)	—
**Household income (US $)**								
	Less than $20,000		Ref		.44		Ref	<.001
	$20,000 to <$35,000		1.27 (0.75-2.17)		—		1.24 (0.79-1.94)	—
	$35,000 to <$50,000		1.28 (0.56-2.89)		—		1.66 (1.08-2.54)	—
	$50,000 to <$75,000		1.95 (0.75-5.08)		—		1.73 (1.17-2.55)	—
	$75,000 or more		2.14 (0.80-5.69)		—		2.81 (1.88-4.22)	—
**Cardiovascular disease**								
	No		Ref		<.001		Ref	<.001
	Yes		2.55 (1.52-4.30)		—		1.44 (1.20-1.72)	—
**Depression**								
	No		Ref		.18		Ref	.63
	Yes		1.34 (0.87-2.05)		—		1.05 (0.85-1.30)	—
**Internet access diversity**								
	One way to access internet		Ref		<.001		Ref	<.001
	More than one way to access		2.72 (1.69-4.38)		—		1.47 (1.29-1.68)	—

^a^OR: odds ratio.

^b^Not available.

**Table 5:** The odds ratio of predictors for social media for health information among Medicaid and non-Medicaid participants.

Variable		Medicaid				Non-Medicaid		
		OR^a^ (95% CI)		*P* value		OR (95% CI)		*P* value
**Year**								
	2017		Ref		.07		Ref	.13
	2018		1.79 (1.04-3.10)		—^b^		1.08 (0.87-1.35)	—
	2019		1.95 (1.05-3.63)		—		1.19 (1.00-1.43)	—
**Gender**								
	Male		Ref		.18		Ref	<.001
	Female		1.36 (0.86-2.14)		—		1.50 (1.30-1.72)	—
**Race**								
	Non-Hispanic White		Ref		.06		Ref	<.001
	Hispanic		1.37 (0.72-2.63)		—		1.44 (1.13-1.83)	—
	Non-Hispanic Black		1.54 (0.82-2.89)		—		1.30 (0.96-1.75)	—
	Non-Hispanic Other		3.57 (1.28-9.97)		—		1.37 (0.90-2.08)	—
	Non-Hispanic Asian		1.86 (0.82-4.24)		—		1.98 (1.39-2.80)	—
**Education**								
	Less than high school		Ref		.21		Ref	.09
	High school graduate		0.70 (0.32-1.51)		—		1.01 (0.54-1.91)	—
	Some college		0.95 (0.45-2.04)		—		1.39 (0.83-2.33)	—
	College graduate or higher		1.27 (0.59-2.72)		—		1.39 (0.83-2.35)	—
**Age group (years)**								
	18-24		Ref		.06		Ref	<.001
	25-44		1.29 (0.61-2.73)		—		0.87 (0.58-1.32)	—
	45-64		1.45 (0.68-3.06)		—		0.57 (0.39-0.84)	—
	65+		0.59 (0.23-1.50)		—		0.30 (0.21-0.42)	—
**Census region**								
	Northeast		Ref		.16		Ref	.09
	Midwest		1.96 (1.03-3.72)		—		0.98 (0.76-1.28)	—
	South		1.73 (0.92-3.26)		—		1.26 (0.99-1.61)	—
	West		1.67 (0.88-3.15)		—		1.24 (0.96-1.59)	—
**Residency**								
	Urban		Ref		.44		Ref	.16
	Rural		0.71 (0.29-1.75)		—		0.81 (0.59-1.09)	—
**Household income (US $)**								
	Less than $20,000		Ref		.97		Ref	.54
	$20,000 to <$35,000		0.96 (0.50-1.87)		—		1.42 (0.94-2.16)	—
	$35,000 to <$50,000		1.15 (0.67-1.95)		—		1.31 (0.87-1.98)	—
	$50,000 to <$75,000		0.94 (0.42-2.11)		—		1.17 (0.84-1.64)	—
	$75,000 or more		0.99 (0.45-2.16)		—		1.22 (0.87-1.72)	—
**Cardiovascular disease**								
	No		Ref		.85		Ref	.47
	Yes		1.04 (0.68-1.58)		—		1.07 (0.89-1.29)	—
**Depression**								
	No		Ref		.51		Ref	<.001
	Yes		1.18 (0.71-1.97)		—		1.54 (1.28-1.86)	—
**Internet access diversity**								
	One way to access internet		Ref		.004		Ref	.01
	More than one way to access		1.98 (1.26-3.11)		—		1.33 (1.09-1.61)	—

^a^OR: odds ratio.

^b^Not available.

## Discussion

According to Ambrosi and colleagues [33], the digital divide can be operationalized in multiple forms. The most common way of defining the digital divide relates to the inequalities of ICT access, for example, complete lack of access to the internet or a smart device. Findings from this study suggest that the Medicaid population is at higher odds of having no access to the internet when compared to the non-Medicaid population. Though worthy of attention and immediate addressing, the elimination or significant reduction of this type of disparity will be an undertaking that will likely not happen in the near future. The second form of the digital divide is caused by different use patterns among individuals who already have access to ICT. An example is people who only use the basic functions (talk and texting) within a smartphone that has many more available features. The key objectives of our study focused on the latter scenario and aimed at assessing the profiles and patterns of digital inequality between the Medicaid and the non-Medicaid populations with some form of internet access on a national level in the United States. We found that the Medicaid population had lower adoption on HIM and MHSR than the non-Medicaid population, which is consistent with a previous study about the effect of socioeconomic status [34]. According to Venkatesh and colleagues' unified theory of acceptance and use of technology (UTAUT), effort expectancy (degree of ease of use of technology) and social influence are important direct factors that are positively associated with the intention and behavior of consumers' technology adoption [35]. Underserved populations like that of Medicaid beneficiaries may perceive eHealth tools to be a more challenging resource to adopt due to lower education as well as eHealth literacy, the ability to access, process, understand, and use the features of the technology [36]. Social influence may also play a critical role in limiting Medicaid participants' use of smart devices to full capacity, though the effect of social influence has not been widely tested among the Medicaid population. Interestingly, we found that the Medicaid population was more likely to use social media to engage in health-related activities. The barriers for underserved communities to regularly access medical resources may push Medicaid participants to use social media as a cost-effective alternative source of medical information. This increased adoption of social media use among the Medicaid population could be a potential double-edged sword. On the one hand, the low quality of medical information on social media may mislead patients to avoid seeking treatment from regular health professionals. Conversely, well-designed social media interventions that specifically target underserved populations could be developed to promote eHealth technology adoption and eHealth literacy through social media. The challenge of this approach is that current social media artificial intelligence and machine learning algorithms may not have the ability to differentiate social media posts that are beneficial and detrimental to users' understanding of health. Therefore, increased exposure to social media interventions may also increase exposure to misleading social media posts.

Several studies have examined the predictors of eHealth tasks or tools adoption among the general population [8,34,37]. Being female, being younger, and having higher income was consistently associated with higher eHealth adoption in general populations, which is consistent with our results based on the non-Medicaid population. However, these factors were not significantly associated with any of the three major eHealth categories: HIM, MHSR, and SMHI. The attenuation of these factor effects in underserved populations was also reported in several studies [38,39], but none has compared the factor effects of underserved populations to their counterparts, nor have they explained the reason. It is possible that the use of eHealth tools among underserved populations like Medicaid beneficiaries was determined by other key factors (eg, effort expectancy and social influence), of which the levels do not vary much across subgroups of gender, age, or income level. Unlike income, attained education was significantly associated with both HIM and MHSR, and the effects were not monotonically increasing with higher education level. Therefore, education may not be the factor directly influencing eHealth tools adoption among this population, or the effect of education was confounded by hidden factors. Medicaid beneficiaries with CVD risk were significantly more likely to engage in HIT and use an eHealth app for health benefits. On the other hand, Medicaid participants with depression demonstrated significantly higher odds only in HIM. The adoption of MHSR involves more self-discipline and motivation, which might be a hurdle for depressive Medicaid respondents to use their smart devices for health-related purposes. This also leaves room for developing interventions or tools to facilitate depressive patients to monitor their mental health and improve their adoption of eHealth tools to monitor their physical health as well. Finally, findings suggest that enhancing internet access diversity was significantly associated with improving HIM, MHSR, and SMHI among both Medicaid and non-Medicaid groups. The odds ratios of shifting internet access from a single source to multiple sources were almost doubled among the Medicaid population compared to their counterparts. Internet access diversity, aligned with infrastructure and supports, may determine the facilitating conditions which directly influence the technology use behaviors based on UTAUT [35]. Thus, providing underserved populations with more sources to access the internet or smart devices (eg, cheap 4G data plan, free public WiFi hot spot, affordable Android smartphone) could be a cost-effective means to improve their eHealth adoption and use. In fact, the Lifeline program, which has been providing discounts on phone service for low-income consumers starting in 1985, has adopted broadband (3G) as a support service since 2016 [40]. As such, increasing awareness of the Federal Communications Commission's Lifeline program is an immediate short-term goal to increase access to smartphones and broadband internet for families eligible for Medicaid.

We believe our study has several strengths. To our best knowledge, this study is the first to investigate eHealth technology and tools adoption among underserved populations, especially the Medicaid enrollees, at the national level in the United States. Therefore, sample selection bias was greatly reduced compared to studies that recruited a small sample size of local participants [38,39]. Additionally, we assessed eHealth technology and tools adoption using seven composite scores constructed from 19 items, which provides a comprehensive picture and reduces measurement errors from using single items. Finally, providing increased attention on this topic will hopefully open a new area of research that may have profound effects on the nation's health and economy. Given the above important contributions to the literature, we acknowledge the limitations of the study. Like other studies using HINTS data sets, our study is based on cross-sectional surveys. Therefore, it could not account for all confounding variables or evaluate causal relationships. The questionnaires designed by HINTS were not suitable to investigate the psychometric constructs that impact eHealth technology adoption directly. In addition, the HINTS data are based on self-reported data from respondents. We are unable to confirm if their answers to the eHealth-related questions were without errors. We found a small portion of respondents reported never accessing the internet, but they also reported a positive response to eHealth technology utilization, though we have excluded those respondents to eliminate spuriousness.

To effectively reduce the disparities in eHealth tools adoption between underserved populations and their counterparts, certain factors must be taken into account when developing interventions or infrastructures for Medicaid beneficiaries. Our study offers initial insights into the factors among the nation’s underserved population. Nevertheless, the fundamental drivers of eHealth use among the Medicaid population may not be fully revealed yet. In addition to confirming this study's findings, future studies should take our investigation to a more granular level and examine the intention and behaviors of eHealth tools utilization among underserved populations under the framework of existing models such as UTAUT. Additionally, the effects of interventions aimed at improving eHealth utilization (eg, improving internet access diversity) could be studied more closely to further validate our findings.

## Appendix

Multimedia Appendix 1 eHealth technologies and tools adoption outcomes and definitions.

Multimedia Appendix 2 Definitions of independent variables.

Multimedia Appendix 3 The number and weighted percentage of respondents with no Internet access, one way to access Internet and multiple ways to access Internet.

Multimedia Appendix 4 Odds ratio (OR) and 95% confidence interval (CI) of Medicaid insurance status by the base model and adjusted model.

## Abbreviations

CVD: cardiovascular disease
EHR: electronic health record
ePHI: electronic personal health information
HHS: US Department of Health and Human Services
HIM: health information management
HINTS: Health Information National Trends Survey
HIT: health information technology
ICT: information and communication technologies
MHSR: mobile health for self-regulation
OPPC: online patient-provider communication
RO: research objective
SMHI: social media for health information
UTAUT: unified theory of acceptance and use of technology
