# Supplementary material for: Patterns and Influencing Factors of eHealth Tools Adoption Among Medicaid and Non-Medicaid Populations From the Health Information National Trends Survey (HINTS) 2017-2019: Questionnaire Study
Source: J Med Internet Res. 2021 Feb 18;23(2):e25809. doi: 10.2196/25809 (PMC7932842; doi:10.2196/25809)
Supplement: Multimedia Appendix 1 [file jmir_v23i2e25809_app1.docx]

**Multimedia Appendix 1**. eHealth technologies and tools adoption outcomes and definitions.

| **Names of composite scores** | **Questionnaire Items** |
| --- | --- |
| Online Patient-provider Communication (OPPC) | In the past 12 months have you used a computer, smart phone, or other electronic means to use email or the Internet to communicate with a doctor or a doctor’s office |
|  | Has your tablet or smartphone helped you in discussions with your health care provider? |
|  | In the past 12 months have you used your online medical record to securely message health care provider and staff (for example, email)? |
| Health information Management (HIM) | In the past 12 months have you used a computer, smart phone, or other electronic means to track health care charges and costs? |
|  | In the past 12 months have you used a computer, smart phone, or other electronic means to look up medical test results? |
| Mobile health for self-regulation (MHSR) | On your tablet or smartphone, do you have any apps related to health and wellness? |
|  | In the last 12 months, have you used an electronic medical device to monitor or track your health? For example, a glucometer or  digital blood pressure device. |
|  | Has your tablet or smartphone helped you track progress on a health-related goal, such as quitting smoking, losing weight, or  increasing physical activity? |
| Social media for health information (SMHI) | In the last 12 months, have you used the Internet to participate in an online forum or support group for people with a similar  health or medical issue? |
|  | In the last 12 months, have you used the Internet to watch a health-related video on YouTube? |
|  | In the last 12 months, have you used the Internet to share health information on social networking sites, such as Facebook or  Twitter? |
| Sharing Information (SI) | Have you shared health information from either an electronic monitoring device or smartphone with a health professional within  the last 12 months? |
|  | Have you electronically sent your medical information to another health care provider? |
|  | Have you electronically sent your medical information to family member or another person involved with your care? |
|  | Have you electronically sent your medical information to a service or app that can help manage and store your health information? |
| Buy or Refill medicine (BRM) | In the past 12 months have you used your online medical record to request a refill of medications? |
|  | In the past 12 months have you used a computer, smart phone, or other electronic means to buy medicine or vitamins online? |
| Decision making (DM) | In the past 12 months have you used your online medical record to help you make a decision about how to treat an illness  or condition? |
|  | Has your tablet or smartphone helped you make a decision about how to treat an illness or condition? |
