# Supplementary material for: Patterns and Influencing Factors of eHealth Tools Adoption Among Medicaid and Non-Medicaid Populations From the Health Information National Trends Survey (HINTS) 2017-2019: Questionnaire Study
Source: J Med Internet Res. 2021 Feb 18;23(2):e25809. doi: 10.2196/25809 (PMC7932842; doi:10.2196/25809)
Supplement: Multimedia Appendix 2 [file jmir_v23i2e25809_app2.docx]

**Multimedia Appendix 2**. Definitions of independent variables.

| **Independent variables** | **Questionnaires items** |
| --- | --- |
| Medicaid status | Do you have Medicaid, Medical Assistance, or any kind of government-assistance plan for those with low incomes or a disability? (Yes/No) |
| Internet access diversity | When you use the Internet, do you access it through a regular dial-up telephone line? |
|  | When you use the Internet, do you access it through broadband such as DSL, cable or FiOS? |
|  | When you use the Internet, do you access it through a cellular network (i.e., telephone, 3G/4G)? |
|  | When you use the Internet, do you access it through a wireless network (WiFi)? |
| Cardiovascular disease (CVD) risk | Has a doctor or other health professional ever told you that you had high blood pressure or hypertension |
|  | Has a doctor or other health professional ever told you that you had a heart condition such as heart attack, angina or  congestive heart failure? |
|  | Has a doctor or other health professional ever told you that you had chronic lung disease, asthma, emphysema, or chronic  bronchitis? |
| Depression | Has a doctor or other health professional ever told you that you had depression or anxiety disorder? |
|  | **Categories** |
| Gender | Male, female |
| Age | 18-24, 25-44, 45-64, and 65 years and older |
| Race/ethnicity | Hispanic, non-Hispanic White, non-Hispanic Black, Asian, Other |
| Residency | Urban, rural |
| US Census region | Northeast, Northwest, South, West |
| Annual household income | less than $20K, $20K-$34.9K, $35K-$49.9K, $50K-$74.9K, and $75K and more |
| Education | Less than high school, High school graduate, Some college, College graduate or higher |
