# Supplementary material for: Patterns and Influencing Factors of eHealth Tools Adoption Among Medicaid and Non-Medicaid Populations From the Health Information National Trends Survey (HINTS) 2017-2019: Questionnaire Study
Source: J Med Internet Res. 2021 Feb 18;23(2):e25809. doi: 10.2196/25809 (PMC7932842; doi:10.2196/25809)
Supplement: Multimedia Appendix 3 [file jmir_v23i2e25809_app3.docx]

**Multimedia Appendix 3.** The number and weighted percentage of respondents with no Internet access, one way to access Internet and multiple ways to access Internet.

|  | 2017 | | | 2018 | | | 2019 | | | |
| --- | --- | --- | --- | --- | --- | --- | --- | --- | --- | --- |
| Internet access diversity | Medicaid (n=499) | Non-Medicaid (n=2747) | P-value | Medicaid (n=503) | Non-Medicaid (n=2936) | P-value | Medicaid (n=796) | Non-Medicaid (n=4547) | P-value |  |
| None | 213 (30.8) | 518 (15.9) | <.001 | 203 (31.5) | 531 (14.2) | <.001 | 283 (24.8) | 755 (13.2) | <.001 |  |
| One way to access Internet | 111 (20.9) | 899 (27.7) |  | 133 (24.9) | 1009 (30.0) |  | 200 (21.1) | 1381 (25.4) |  |  |
| More than one way to access | 174 (48.3) | 1329 (56.4) |  | 166 (43.6) | 1396 (55.8) |  | 303 (54.0) | 2380 (61.4) |  |  |
