# Supplementary material for: Patterns and Influencing Factors of eHealth Tools Adoption Among Medicaid and Non-Medicaid Populations From the Health Information National Trends Survey (HINTS) 2017-2019: Questionnaire Study
Source: J Med Internet Res. 2021 Feb 18;23(2):e25809. doi: 10.2196/25809 (PMC7932842; doi:10.2196/25809)
Supplement: Multimedia Appendix 4 [file jmir_v23i2e25809_app4.docx]

**Multimedia Appendix 4**. Odds ratio (OR) and 95% confidence interval (CI) of Medicaid insurance status by the base model and adjusted model.

|  | Unadjusted | | Adjusted ^a^ | |
| --- | --- | --- | --- | --- |
| Outcome | Odds ratio (%95 CI) | P value | Odds ratio (%95 CI) | P value |
| Decision making (DM) | 1.05 (0.81-1.37) | 0.683 | 1.00 (0.74-1.33) | 0.982 |
| Health information Management (HIM) | 0.50 (0.38-0.66) | <.001 | 0.64 (0.50-0.82) | <.001 |
| Buy or refill medicine (BRM) | 0.66 (0.52-0.82) | <.001 | 0.78 (0.60-1.01) | 0.058 |
| Mobile health for self-regulation (MHSR) | 0.62 (0.53-0.73) | <.001 | 0.63 (0.53-0.76) | <.001 |
| Sharing Information (SI) | 0.97 (0.69-1.36) | 0.848 | 1.15 (0.82-1.60) | 0.406 |
| Social media for health information (SMHI) | 1.49 (1.23-1.80) | <.001 | 1.35 (1.12-1.63) | 0.002 |
| Online patient-provider Communication (OPPC) | 0.72 (0.58-0.90) | 0.004 | 0.90 (0.70-1.16) | 0.409 |

| ^a^ “Adjusted model” included gender, race, education, age group, census region, residency and internet  access diversity as covariates. |
| --- |
